# Micro-Coring: A Novel Approach to Perioral Rejuvenation

**DOI:** 10.1093/asj/sjae120

**Published:** 2024-05-31

**Authors:** Katherine H Carruthers, Krishna Vyas, Katya Remy, Justin C McCarty, William G Austen

## Abstract

**Background:**

A long philtrum and poor perioral skin quality are stigmata of the aging face. Micro-Coring is a novel technology that allows for scarless skin removal.

**Objectives:**

In this study we aimed to determine whether micro-coring can shorten the philtrum and improve perioral skin quality.

**Methods:**

A retrospective cohort study was performed on patients who underwent facelift with perioral micro-coring and age- and BMI-matched controls who underwent facelift alone. Preoperative and postoperative 3-dimensional facial imaging was performed. Standard perioral distances and percentage of change were calculated. Perioral skin quality was evaluated by blinded raters with the Scientific Assessment Scale of Skin Quality (SASSQ) and Global Aesthetic Improvement Scale (GAIS).

**Results:**

Thirteen patients and 13 controls were included, with a mean follow-up of 8.9 months (range 3.0-21.5). Patients had significantly shorter mean philtrum length postoperatively compared to preoperatively, with an average decrease of 6.18% (±2.25%; *P* < .05). Controls did not experience significant changes in philtrum length (*P* > .05). There were no significant changes in other perioral measurements. Perioral skin elasticity and wrinkles significantly improved in patients compared to controls and patients had significantly greater GAIS scores (*P* < .05).

**Conclusions:**

Micro-Coring can achieve perioral rejuvenation through measurable shortening of the philtrum and observable improvement in skin quality. Nonsurgical techniques continue to find new ways to achieve aesthetic goals without significant recovery or scarring and offer value to patients and clinicians.

**Level of Evidence: 3:**

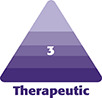

Lengthening of the philtrum and the development of perioral rhytids are common hallmarks of facial aging.^1-3^ Current non-surgical and surgical facial rejuvenating treatments have known limitations. Fillers and botulinum toxin are limited by their short-term effect, microneedling does not result in actual skin removal, and laser resurfacing carries the risk of thermal injury and adverse skin reactions such as dyspigmentation.^4,5^ There are no studies to date that describe whether these procedures result in skin tightening or shortening of the philtrum length.

Although surgical techniques generally provide more definitive and longer-lasting results, they are limited by prolonged recovery and scarring.^6-8^ The lip lift effectively shortens the upper lip through the excision of an ellipse of skin at the cephalad portion of the philtrum.^9,10^ However, adverse scarring has been reported in up to 7.23% of patients.^9-12^ Further, although facelift surgery provides the most pronounced rejuvenating outcomes, incongruity frequently persists due to the continued presence of an elongated philtrum and poor perioral skin quality, because these concerns are not typically addressed with this surgery.^13,14^

Micro-Coring is a novel procedure developed for minimally invasive facial rejuvenation with the Ellacor device (Cytrellis Biosystems, Inc., Woburn, MA).^15^ The system utilizes modified hollow hypodermic needles to remove <0.5-mm-diameter full-thickness microcores of skin. These needles are similar in principle to punch biopsies and can remove between 5% to 8% of the skin's surface area. Because the size of the cores removed is below the threshold known to induce scarring, treated areas undergo a regenerative healing process rather than a repair process.^16,17^ This results in scarless skin removal and an organized increase in collagen and elastin content, epidermal and papillary dermal thickening, and restoration of rete ridges.^16,17^ Micro-Coring has been shown to be safe and effective for skin tightening and wrinkle reduction in multiple clinical trials.^15,18^

When applied to the perioral region, micro-coring may provide longer-term rejuvenation than traditional nonsurgical treatments and does not carry the risk of thermal injury associated with laser resurfacing treatments. Furthermore, because no incisions are required, micro-coring may also result in shorter recovery periods than surgical treatments.^5,15^ In this study, we aimed to determine whether the skin changes that occur with micro-coring applied for lower face rejuvenation can result in shortening of the philtrum, changes in other perioral measurements, and improvement in perioral skin quality.

## METHODS

### Study Design

We performed a retrospective matched cohort study of patients who underwent micro-coring with facelift compared to controls who underwent facelift alone, evaluating the effect on philtrum length and skin quality. The study was reviewed and approved by the Institutional Review Board of the Massachusetts General Hospital in Boston, Massachusetts (protocol 2023P002263).

### Participants

Consecutive patients who underwent perioral micro-coring combined with a facelift for facial rejuvenation between January 2021 and June 2023 were included. Age-matched and BMI-matched controls who underwent facelift surgery without micro-coring during the same treatment period were also identified. If the patient raised concern for perioral aging and examination revealed perioral rhytids, the option to undergo micro-coring at the time of facelift surgery was discussed during the preoperative consultation. Patients were informed about the expected outcomes, recovery, risks and benefits, and cost of micro-coring. Because micro-coring is typically performed as an adjunct to facelift surgery, and patients who undergo facelift routinely undergo standardized preoperative and postoperative facial imaging at our institution, facelift patients were chosen as controls.

All interventions were performed by Dr Austen at a single institution. Inclusion criteria were females and males over 40 years old with Fitzpatrick skin types I through IV. Patients were included if they received both preoperative and postoperative 3-dimensional (3D) imaging following a single treatment. Exclusion criteria included additional perioral nonsurgical or surgical intervention in the treatment areas during the follow-up period, including filler or botulinum toxin, dermabrasion, laser, microneedling, radiofrequency, and chemical or mechanical peels. Additional exclusion criteria included absence of preoperative and postoperative imaging.

### Operative Technique

**Figure 1.**
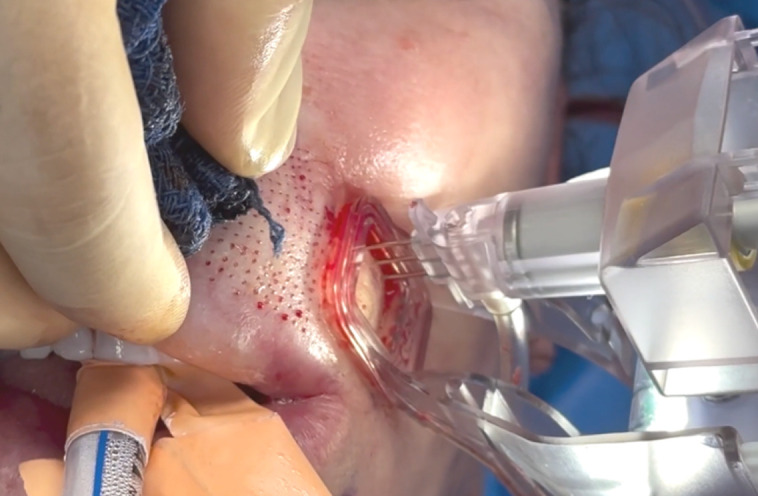
Perioral micro-coring procedure in a 60-year-old female patient. The handheld device contains hollow needles that are inserted into the skin across the treatment area.

Micro-Coring was performed with the Ellacor device. The device is composed of a handpiece connected to a system control. The handpiece is reusable and can be covered in a sterile drape. It contains consumable hollow needles and an aspiration system. The operator moves the handpiece across the treatment area (Figure 1). The percentage of desired tissue removal and the depth of penetration into the skin can be selected by the operator. In this study, micro-coring settings ranged from 5% to 8% skin surface area removal and a 2.5- to 4-mm coring depth. The device is commercially available and was US FDA-approved in 2021 for the treatment of moderate to severe wrinkles in the mid and lower face of patients with Fitzpatrick skin types I to IV.^15^

**Figure 2.**
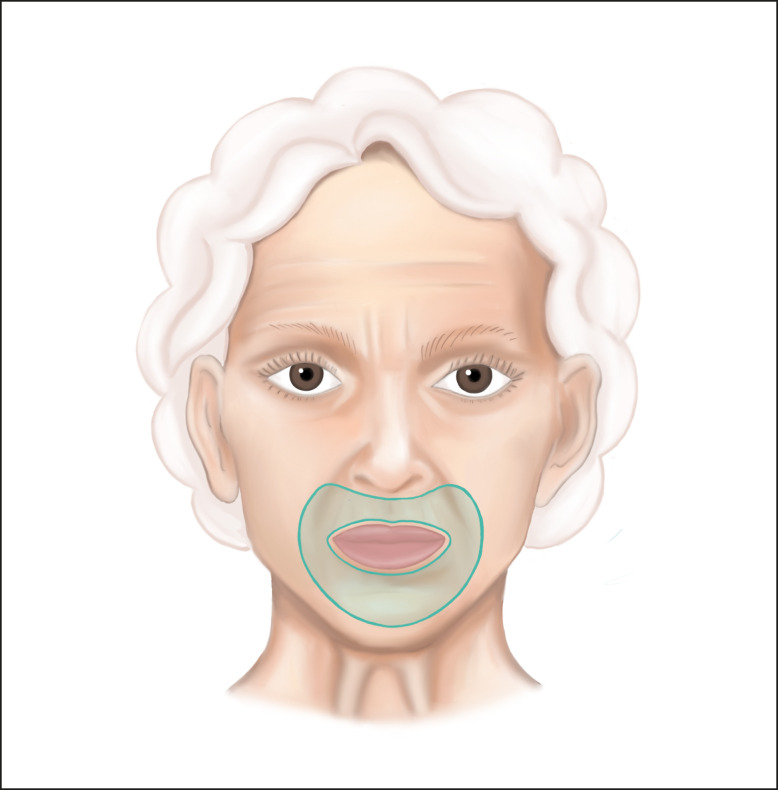
Perioral micro-coring treatment area. The treatment area involves the upper and lower lip subunits and is delineated laterally by the nasolabial fold and marionette lines and medially by the vermillion. © 2024 Katya Remy. Used with permission.

In this study, the micro-coring procedure was performed similarly, as a single treatment session for all treated patients as previously described.^18^ The perioral area was treated as illustrated in Figure 2. Treatments typically took between 15 and 20 minutes and were performed in the operating room under general anesthesia following a standard superficial musculo-aponeurotic system (SMAS) plication facelift. Injectable local anesthetic was administered before the procedure based on standard procedures and at the physician's discretion. Typically, patients were injected with approximately 20 to 40 mL of a 1% lidocaine and epinephrine solution for postoperative pain, hemostasis, and turgor (see Video, demonstrating the micro-coring procedure performed intraoperatively). Patients undergoing micro-coring were counseled about potential postoperative hyperemia in the treatment area, which can last approximately 2 to 3 weeks.

### Assessment of Perioral Measurements

All patients had preoperative and postoperative 3D facial imaging with the VECTRA XT 3D Imaging System (Canfield Scientific, Inc., Fairfield, NJ) as part of standard care by Dr Austen. VECTRA imaging provided validated and standardized stereophotogrammetry.^19^ The VECTRA Analysis Module (VAM) software (Canfield Scientific) was employed to review, measure, and compare images. Preoperative baseline images were registered to the 3D axis grid and compared to subsequent postoperative imaging. Comparative images were viewed side-by-side, and landmarks were placed in similar locations on each image to register subsequent images to the baseline image.

**Figure 3.**
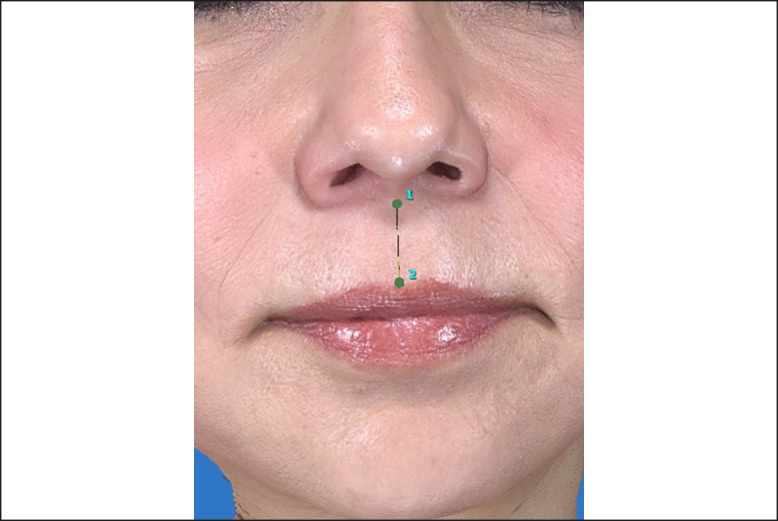
Calculation of philtrum length. Three-dimensional imaging analysis software was utilized to determine the primary outcome measurement, which was the philtrum length. Philtrum length was measured by calculating the distance from subnasale (point 1) to cupid's bow nadir (point 2). This is the case of a 59-year-old female patient.

Once images were registered to each other, perioral outcome measurements were obtained by 2 independent study staff blinded to each other's measurements. The philtrum length was measured by computerized calculation of the distance from the subnasale to cupid's bow nadir (see Figure 3). The differences between postoperative and preoperative values were calculated to determine the difference and percentage reduction in length. The average measurements of the 2 study staff were calculated for analysis. Other measurements included upper lip height, upper lip length, lower lip height, lower lip length, and lip to menton distance.

### Assessment of Skin Quality

Ten plastic surgeons assessed perioral skin quality of all patients and controls with photographs that were presented consecutively and in random order. Randomization was performed with an online list randomization tool (RANDOM.ORG, Dublin, Ireland) after the images were assigned reference numbers. While being blinded to the intervention as well as preoperative or postoperative status, raters completed the Scientific Assessment Scale of Skin Quality (SASSQ), assessing perioral loss of elasticity, wrinkles, skin surface roughness, lentigines/pigmentation, erythema, blemishes, and pore size with a 5-point Likert scale (0 = none, 1 = mild, 2 = moderate, 3 = severe, 4 = very severe).^20^ Raters then assessed the degree of skin quality improvement of consecutive side-by-side before-and-after photographs with the Global Aesthetic Improvement Scale (GAIS) (1 = very much improved, optimal cosmetic result; 2 = much improved, marked improvement in appearance from the initial condition, but not completely optimal; 3 = improved, obvious improvement in appearance from the initial condition; 4 = no change, the appearance was essentially the same as baseline; 5 = worse, the appearance was worse than the original condition).^15^

### Statistical Analyses

Patients undergoing micro-coring with facelift and controls undergoing facelift without micro-coring were matched by age (±10 years) and BMI (±5 kg/m2) at the time of the procedure. A final 1:1 matching rate was the goal for analysis. Descriptive statistics were reported in means and standard deviations or median and range, depending on normality. Paired *t* tests were conducted to compare preoperative with postoperative values as well as patients with controls. Significance was defined as a 2-sided *P* < .05. Analyses were computed with Stata version 17 (StataCorp L.P., College Station, TX).

## RESULTS

### Patient Cohort

A total of 13 micro-coring patients and 13 age- and BMI- matched controls were included in the final analysis. There were 24 (92.3%) females and 2 (7.7%) males. The mean age was 64.7 (±7.4, range 53.2-82.0) years, and mean BMI was 23.6 (±5.2) kg/m2. All patients had Fitzpatrick skin types I to IV. The average coring surface area was 7% (±1.5%) per patient. The mean follow-up was 8.9 months (range 3.0-21.5). No patients developed postoperative hematoma, skin necrosis, infection, or wound dehiscence requiring revisional surgery or reintervention. There were no cases of prolonged hyperemia or dyspigmentation (hyperpigmentation or hypopigmentation) following micro-coring. See Table 1 for patient demographics.

**Table 1.** Patient Demographics

Variable	Micro-Coring patients	Controls	*P* value
	*n* = 13	*n* = 13	
Age, years; mean (SD)	64.5 (5.2)	67.5 (8.1)	.38
BMI, kg/m2; mean (SD)	23.6 (3.3)	22.8 (3.8)	.65
Gender, *n* (%)			
Female	12 (92.3)	12 (92.3)	1.00
Male	1 (7.7)	1 (7.7)	1.00
Philtrum length, mm; mean (SD)	17.5 (2.3)	17.2 (1.6)	.68

BMI, body mass index; SD, standard deviation.

### Perioral Measurements

**Figure 4.**
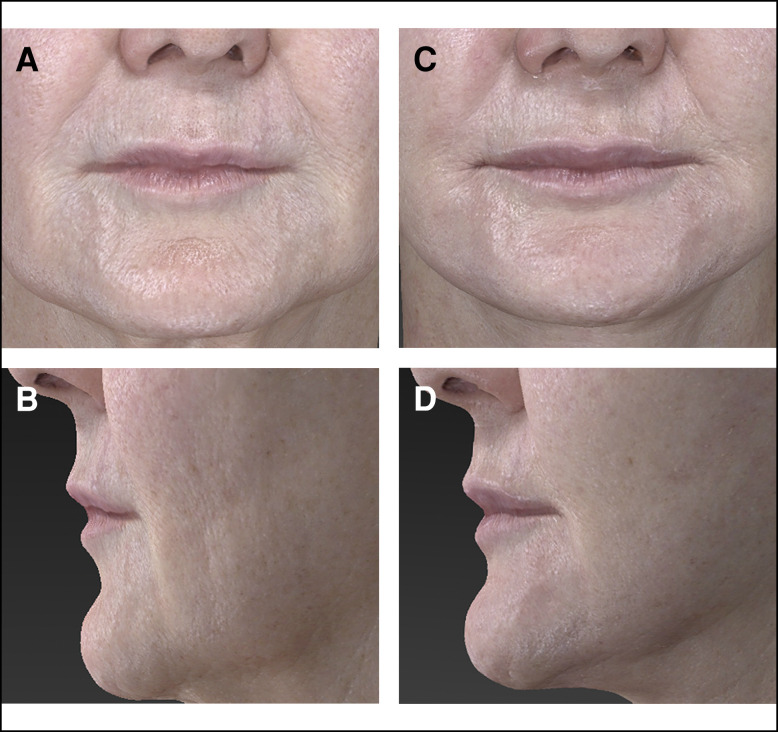
A 65-year-old female patient underwent face and neck lift surgery with perioral micro-coring set at 5-8% coring density. Preprocedure imaging (A and B) demonstrates a philtrum length of 19.20 mm. Four months postprocedure imaging (C and D) demonstrates a philtrum length of 18.15 mm (5.45% reduction from preprocedure).

**Figure 5.**
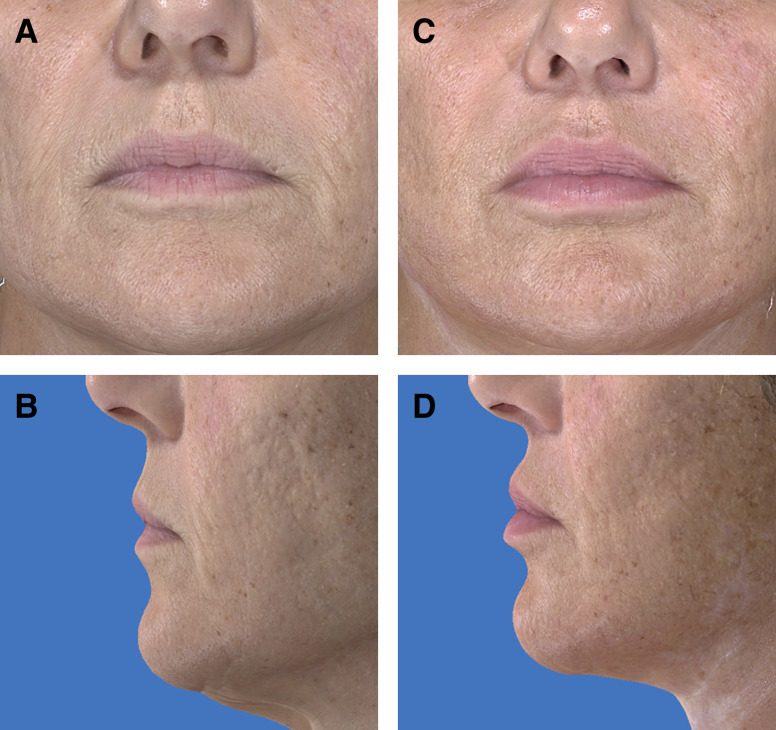
A 66-year-old female patient underwent face and neck lift surgery with perioral micro-coring set at 8% coring density. Preprocedure imaging (A and B) demonstrate a philtrum length of 14.94 mm. Sixteen months postprocedure imaging (C and D) demonstrate a philtrum length of 13.71 mm (8.43% reduction as compared to preprocedure).

Preoperative philtrum length was statistically similar between micro-coring patients and facelift controls (17.5 ± 2.3 mm vs 17.2 ± 1.6 mm, *P* > .5). Patients treated with micro-coring had statistically significantly shorter mean philtrum lengths postoperatively than preoperatively (16.4 mm vs 17.5 mm, *P* < .0001). The average decrease in philtrum length was 6.18% (±2.25%), with an average shortening of 1.07 mm (±0.39 mm). Controls did not experience statistically significant changes in mean philtrum length postoperatively compared to preoperative measurements (17.3 mm vs 17.2 mm, *P* > .1; see Tables 2, 3). The postoperative philtrum length in micro-coring patients was found to be significantly shorter than that of controls (*P* < .05). There were no statistically significant changes in any of the other recorded perioral measurements. See Figures 4 and 5 for preoperative and postoperative photographs demonstrating philtrum shortening following micro-coring.

**Table 2.** Micro-Coring Cases

Ellacor patients	Pre-op philtrum height (mm)	Post-op philtrum height (mm)	Change in height (mm)	% Change	Coring % (per square cm)	Follow-up time (months)
1	19.61	18.22	−1.39	−7.07	8	3.3
2	15.21	14.27	−0.94	−6.17	5	3.9
3	16.34	15.77	−0.56	−3.44	5	4.4
4	19.20	18.15	−1.05	−5.45	5–8	4.6
5	20.13	18.53	−1.60	−7.95	5	5.6
6	17.21	16.45	−0.76	−4.42	8	5.9
7	14.24	12.81	−1.43	−10.05	8	6.3
8	18.94	17.85	−1.09	−5.76	8	7.8
9	15.28	14.68	−0.60	−3.93	8	7.8
10	14.94	13.71	−1.23	−8.43	8	16.0
11	21.94	20.94	−1.00	−4.56	8	10.7
12	16.57	15.97	−0.60	−3.62	8	11.4
13	18.02	16.30	−1.72	−9.54	8	21.5

**Table 3.** Control Cases

Controls	Pre-op philtrum height (mm)	Post-op philtrum height (mm)	Change in height (mm)	% Change	Follow-up time (months)
1	17.18	18.02	0.84	4.89	3.0
2	16.96	17.33	0.37	2.18	3.1
3	18.79	18.59	−0.2	−1.06	5.3
4	18.18	18.25	0.07	0.39	5.6
5	17.67	17.43	−0.24	−1.36	7.9
6	17.28	18.13	0.85	4.92	9.8
7	16.86	16.19	−0.67	−3.97	10.1
8	15.53	15.67	0.14	0.9	10.3
9	20.19	20.0	−0.19	−0.94	10.4
10	15.58	15.46	−0.12	−0.77	10.9
11	17.17	17.55	0.38	2.21	13.3
12	13.68	13.59	−0.09	−0.66	15.7
13	18.32	18.14	−0.18	−0.98	17.8

### Perioral Skin Quality

**Figure 6.**
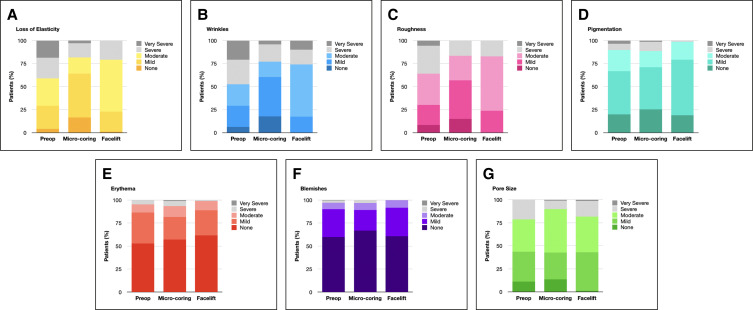
Scientific Assessment Scale of Skin Quality (SASSQ) in micro-coring patients vs facelift-only controls. Micro-Coring patients had significantly better scores for loss of elasticity and wrinkles compared to preoperative values and facelift-only controls (*P* < .05). All other metrics were similar preoperatively and postoperatively in patients and controls (*P* > .05).

**Figure 7.**
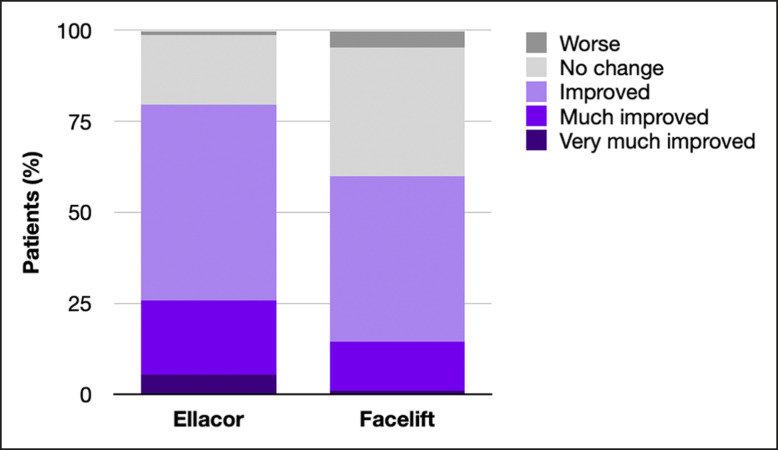
Global Aesthetic Improvement Scale (GAIS) for perioral skin quality in micro-coring patients vs controls. Patients had significantly more improvement in GAIS than controls (79.7% vs 60.0%, *P* < .05).

On the SASSQ, micro-coring patients and facelift controls had statistically comparable preoperative perioral skin quality scores for all assessed metrics (loss of elasticity, wrinkles, roughness, pigmentation, erythema, blemishes, and pore size; *P* > .05). Postoperatively, micro-coring patients had significantly improved mean scores in perioral elasticity and wrinkles (*P* < .05), but not in roughness, pigmentation, erythema, blemishes, or pore size (*P* > .05). Facelift controls had statistically significant improvements in perioral elasticity (*P* < .05), but not in wrinkles, roughness, pigmentation, erythema, blemishes, or pore size (*P* > .05). Patients had statistically significant better ratings in perioral elasticity and wrinkles compared to controls (*P* < .05), but similar roughness, pigmentation, erythema, blemishes, and pore size (*P* > .05) (see Figure 6). Significantly more micro-coring patients had some degree of perioral skin quality improvement on the GAIS when compared to controls (79.7% vs 60.0%, *P* < .05; see Figure 7).

## DISCUSSION

The aim of this study was to evaluate the effects of perioral micro-coring on philtrum length and skin quality. We found that patients who underwent micro-coring achieved statistically significant shortening of their philtrum. The reduction in philtrum length, which ranged between 3.44% and 10.05%, correlated to the percentage of skin surface area removed, which ranged between 5% and 8%. In addition, micro-coring patients achieved significantly better improvements in perioral skin elasticity and wrinkles compared to baseline and compared to facelift controls. However, other skin quality metrics, such as pigmentation, roughness, blemishes, erythema, and pore size did not significantly change.

The clinical results in this study are supported by several preclinical studies describing the skin removal and biological tightening mechanisms of micro-coring.^17,21^ One study showed that porcine skin treated at 10% coring density experienced a reduction in skin surface area by 9%.^21^ This was statistically significant when compared with baseline and a control.^21^ In another animal model, micro-coring demonstrated an 89% increase in newly organized collagen and elastin fibers at 3 months postoperatively.^17^ Rete ridges, which are known to flatten with age, also experienced restoration of undulations. Additionally, it has been suggested that microcores undergo microcontracture, possibly due to myofibroblast activation.^17^ Together, these mechanisms may induce skin tightening and translate into philtrum shortening and visible improvements in perioral skin quality.^16^

The skin tightening mechanisms of micro-coring with its scarless skin removal distinguish micro-coring from other nonsurgical and surgical rejuvenating treatments. Although microneedling can stimulate collagen and elastin synthesis, its effects in achieving philtrum shortening or significant changes in skin tightness and wrinkles may be limited because the skin is only punctured and not removed.^4,22-27^ Accordingly, studies have shown that the mean change in the Lemperle Wrinkle Severity Scale after treatment with microneedling was lower than that after micro-coring.^18,28^ Further, although laser resurfacing is commonly applied to treat wrinkles, the ablated skin can lead to the accumulation of microepidermal necrotic debris and prevent early closure of ablated skin cores. This may limit the amount of skin surface area reduction that can be achieved.^15,29^ Currently, no studies exist that compare skin quality results of micro-coring and laser resurfacing techniques.

The amount of philtrum reduction that can be obtained from a single micro-coring procedure is less than that of a surgical lip lift, which remains the most effective procedure to achieve significant philtrum shortening.^9,10^ However, the amount of philtrum reduction achieved with a single micro-coring procedure may be suitable for patients with only mild or moderate philtrum excess, or for patients who are not candidates for a surgical procedure. Additionally, further philtrum shortening may be achieved over time with multiple micro-coring procedures while avoiding the common limitations associated with the traditional lip lift (prolonged recovery time, risk of adverse scarring) and concomitantly improving skin quality. Micro-Coring also does not preclude having a lip lift at a later date.

Although facelift surgery is the gold standard facial rejuvenating treatment, it is thought that the perioral region is not significantly influenced.^14^ Our study showed that control patients who underwent facelift without micro-coring did not experience significant changes in philtrum length, and most skin quality metrics were not influenced. Therefore, micro-coring may be a beneficial adjunct to achieve more harmonious and youthful outcomes by addressing the perioral region.

The findings of this study must be viewed within the context of its limitations. First, the analysis is limited by its small sample size and retrospective nature. Although previous prospective clinical trials have shown the effectiveness of micro-coring for improvement of skin quality, similar trials would be necessary to confirm the effects of micro-coring on philtrum shortening. Further, the retrospective nature of this study limited our ability to capture data on postoperative hyperemia and other potential adverse events at various follow-up time points. In a previous prospective study, the safety parameters of facial micro-coring were evaluated, including healing profile (presence of ecchymosis, purpura, fluid accumulation, hyperpigmentation, hypopigmentation, roughness, dryness, inflammation, erythema, and crusting).^15^ These were scored 0 (absent), 1 (trace), 2 (mild), 3 (moderate) or 4 (severe). Study findings included trace side effects (roughness, dryness, inflammation) up to day 7, trace redness on days 1 to 15 that was absent on day 30 and subsequently, as well as trace hyperpigmentation on day 30 that resolved by day 90.^15^ These results suggest a low rate of adverse effects. Third, although no patients reported having undergone additional perioral rejuvenating interventions (including lip fillers) during the study period, verified manually with charts, the retrospective design limited the possibility of capturing potential cases in which patients had perioral treatments (such as lip filler) at outside institutions. Moreover, skin quality assessment was limited by photographic evaluation and a lack of objective measures. More objective skin quality assessment tools exist (eg, to evaluate skin elasticity), but were not available for the current analysis.^30^ Further, because our analysis only included individuals with Fitzpatrick scale I to IV skin types, the homogeneity of our study sample limited the generalizability of our findings. The initial trials for FDA approval have been completed for patients with Fitzpatrick skin types I to IV. Finally, we did not evaluate patient reported-outcomes; however, this has been previously reported in a prospective clinical trial investigating 97 micro-coring treated areas, indicating an overall satisfaction rate of 85.6%.^18^ The inclusion of patient-reported outcomes for philtrum shortening will be the basis of future larger studies.

## CONCLUSIONS

Patients undergoing lower face micro-coring for facial rejuvenation may expect a measurable decrease in philtrum length and observable improvement in perioral skin quality. As nonsurgical techniques continue to gain traction, finding new ways to achieve aesthetic goals without considerable recovery or scarring will be of increasing value.

## Supplemental Material

This article contains supplemental material located online at www.aestheticsurgeryjournal.com.

## Supplementary Material

sjae120_Supplementary_Data
